# Does Uterine Fibroid Adversely Affect Obstetric Outcome of Pregnancy?

**DOI:** 10.1155/2018/8367068

**Published:** 2018-03-26

**Authors:** Hend S. Saleh, Hala E. Mowafy, Azza A. Abd El Hameid, Hala E. Sherif, Eman M. Mahfouz

**Affiliations:** Obstetrics and Gynecology Department, Faculty of Medicine, Zagazig University, Zagazig, Egypt

## Abstract

**Background:**

Fibroid is the most common benign tumor of the uterus and if associated with pregnancy may adversely affect the outcome of pregnancy. Objective of the present study was to assess the obstetric outcome (maternal and fetal) in pregnancy with fibroid.

**Methods:**

A prospective observational study was performed over a period from May 2015 to August 2017 at Obstetrics and Gynecology Department in Zagazig University Hospitals, Egypt. 64 pregnant patients with >2 cm fibroid were taken in the study. Routine fundamental investigations were done for all. They were followed during antenatal period clinically and scanned by ultrasonogram which was done at booking visit and during subsequent visits to assess the change in the size of the fibroid and other obstetric complications. Maternal age, parity, size of fibroid, complications during pregnancy, and mode of delivery were noted.

**Results:**

64 pregnant patients with uterine fibroids were recruited; 47 of them completed the study to the end. The average age was 31.80 ± 3.27 years, body mass index (BMI) [calculated as weight in kilograms divided by the square of height in meters] was 24.67 ± 2.46, primigravida was 23.4%, multigravida was 76.6%, duration of menstrual cycle/day was 29.68 ± 3.10, and duration of menstrual period/day was 6.46 ± 1.12. The percentage of spontaneous conception was 59.57% and 40.43% for using assisted reproductive technology. The results of obstetric outcome were spontaneous abortion in 2%, premature delivery in 27.7%, and delivery at 37–41 weeks of pregnancy in 70.2%. The mode of delivery was vaginal delivery in 15% and cesarean sections in 85%. Also, 34% had threatened miscarriage, 21% had preterm labor, 2% had antepartum bleeding in the form of placenta previa, 4% had abdominal pain needing admission, one of them underwent laparotomy and was diagnosed as red degeneration, 2 (4%) had postpartum hemorrhage, and only one needed blood transfusion. Cesarean sections were done in 85%. Neonatal outcome was acceptable with no perinatal mortality. There were no significant differences between patients with single or multiple fibroids as regards the obstetric outcome or type of fibroid either intramural or subserosal. The obstetric outcomes were not significantly affected by the number, size, or type of fibroids.

**Conclusions:**

Even most of fibroids in pregnancy are asymptomatic but may be associated with some complications affecting the course of pregnancy and labor. So, pregnancy has to be cautiously screened in the antenatal period, through regular follow-up, to detect any adverse obstetric complications and so improve the outcome.

## 1. Introduction

Myomas are the most frequently recorded benign smooth muscle tumor of the uterus, affecting 20%–60% of women of reproductive age and may negatively affect fertility and outcome of pregnancy [1]. As most fibroids are asymptomatic, the true prevalence of fibroids may be greatly higher [2]. The incidence of fibroids in pregnancy reported ranges from 0.1 to 10.7% of all pregnancies and increases as the female chooses to postpone pregnancy later on [3]. It was found that 10%–40% of prepartum complications which happened in pregnancy with fibroid have been associated with the presence of it [4].

Myomas have been complicated by changes like degeneration leading to abdominal pain whose severity is varied from mild to acute abdomen [5]. Also, they are related to a lot of ante-, intra-, and postpartum complications like spontaneous abortion, antepartum hemorrhage, placental abruption, malposition of the fetus, fetopelvic disproportion, premature rupture of membranes, retention of the placenta, postpartum hemorrhage (PPH), preterm delivery, low birth weight infants, dysfunctional labor, and increased need to cesarean deliveries [6].

The principal aim of this study was to inspect obstetric outcomes (maternal and fetal) of pregnancies with fibroids and any associated complications. Furthermore, the secondary aim was about the modification of antenatal care of such patients to improve the outcomes.

## 2. Patients and Methods

64 patients signed up in this current prospective observational study. Their age ranged from 22 to 43 years. They were recruited from antenatal clinics at Obstetrics Department in Zagazig University Hospitals with pregnancy with fibroid after attending first-trimester ultrasonography examination which diagnosed them. They underwent both consequent antenatal care and delivery at the study institute in the study time. Ultrasonogram was done at successive visits to evaluate the change in the size of the fibroid and any associated complications either in fibroid or in pregnancy in general. The period of study was from May 2015 to August 2017. Patients with fibroid of ≥2 cm were included in the study. Excluded criteria were history of any surgical manipulation of uterus such as cesarean section, resection of uterine septum or myomectomy, any uterine malformation, adenomyosis (uterine adenomyoma), any general disease, for example, cerebrovascular, cardiovascular, or diabetes mellitus, and renal insufficiency. After the protocol of this study was approved by the research ethics committee of Zagazig University Hospitals, informed consent (verbal and written) was obtained from all participants. Full patient history, clinical examination, and demographic data were recorded. All participants undertook booking ultrasonographic examination then repeated in every antenatal care with detailed obstetric report with comment on any adverse episodes related to characters of fibroid as size, number, place, and so forth). Measurements at 12–16 weeks were used as reference and compared with those which were taken at 22–26 weeks and 28–34 weeks of pregnancy. The information of obstetric occasions throughout antenatal period and delivery were recorded. Observations of patients till the end of puerperal period were also documented. Qualitative variables were expressed as frequency and percentage, compared using the Pearson *χ*^2^ test, continuity correction *χ*^2^ test, and the Fisher exact test. Quantitative variables were expressed as mean ± SD and compared using the Student *t*-test, Mann–Whitney *U* test, analysis of variance, and the paired Student *t*-test. All data were analyzed using SPSS version 17.0 (SPSS Inc., Chicago, IL, USA). *P* < 0.05 was considered statistically significant.

## 3. Results

Present study included 64 women who were having pregnancy with uterine fibroids. Fibroids that are more than or equal to 2 cm were included in the study.

10 patients were excluded due to deviation from the inclusion criteria; three had previous cesarean sections, one had previous myomectomy, one had uterine malformation, one had uterine adenomyosis, and 4 had medical disorder (two had history of diabetes mellitus and one had history of chronic hypertension).

**Figure 1:**
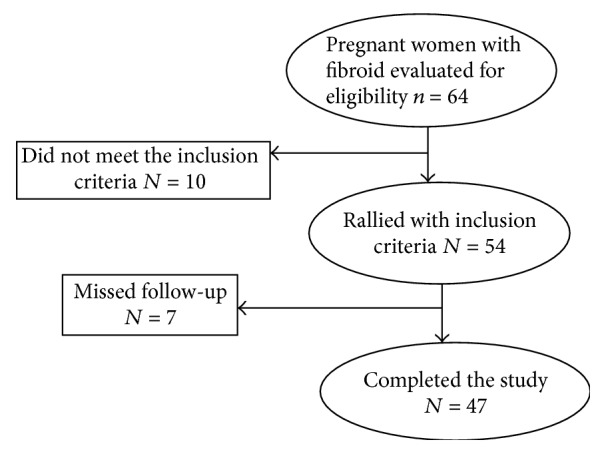
Participants of the study in flow chart.

The follow-up of 7 cases was lost. So, ending data were included from 47 patients only (Figure 1). Ultrasonic examinations of the fibroid at 10–14 weeks, 20–24-weeks, and 28–34 weeks were recorded and studied statistically.

The average of demographic data were as follows: for* age *31.80 ± 3.27 years,* body mass index* (BMI) [calculated as weight in kilograms divided by the square of height in meters] 24.67 ± 2.46,* gravidity *2.63 ± 1.21, parity 1.26 ± 1.03,* duration of menstrual cycle/day *29.68 ± 3.10, and* duration of menstrual period/day *6.46 ± 1.12.

The percentage of spontaneous conception was 59.57% and 40.43% for using assisted reproductive technology (Table 1).

Maternal outcome during antenatal period was represented in Table 2. 16 (34%) had threatened miscarriage (vaginal bleeding occurring at <28 weeks of pregnancy), 10 (21%) had preterm labor, 1 (2%) had antepartum bleeding in the form of placenta previa, 2 (4%) had abdominal pain needing admission, one of them underwent laparotomy and was diagnosed as for red degeneration, 2 (4%) had postpartum hemorrhage (estimated blood loss ≥ 1000 mL for cesarean deliveries or ≥500 mL for vaginal deliveries), and only one needed blood transfusion.  Table 3 showed pregnancy outcome (spontaneous abortion 1 (2%), premature delivery (delivery at 28–<37 weeks of pregnancy) 13 (27.7%), or delivery at 37–41 weeks of pregnancy 33 (70.2%)) and also the mode of delivery {vaginal delivery in 15% or cesarean sections in 85%}. Neonatal outcome represented in Table 4 showed that only one neonate had congenital anomaly in the form of cleft palate. The average fetal weight was 2978.15 ± 374 with good Apgar score with no perinatal mortality. There were no significant differences between patients with single or multiple fibroids as regards the obstetric outcome (Table 5) or type of fibroid either intramural or subserosal (Table 6). The changes in size of fibroid through the pregnancy were represented (Table 7). There was a significant increase in fibroid size only between the 14–16-week and 22–26-week examinations and also between 11–14-week and the 28–34-week examinations when fibroids were below 2 cm in diameter.

## 4. Discussion

The size, number, and type of fibroids had no significant importance with occurrence of adverse outcomes in this current study. This agreed with the study of Klatsky et al. 2008 [7] and study of Poovathi and Ramalingam 2016 [8]. Even Stout et al. 2013 discussed the adverse effect of fibroid on twins pregnancy and also found no significant relations [9]. On general the fibroids did not have significant adverse effects on obstetric outcomes either maternal or neonatal in current study.

Follow-up of the size of fibroid during antenatal period showed a significant increase in size between 14–16 weeks and 22–26 weeks and between 14–16 weeks and 28–34 weeks in fibroids that were ≤3 cm in diameter at the earliest scan and these results were of the same opinion of Wang et al. 2016 [10]. As regards the fibroid type (intramural and subserosal), it was not associated with adverse obstetric outcomes. Size of fibroid has been associated with increased admissions for fibroid pain, postpartum hemorrhage, postpartum blood transfusions, or increased blood loss in some studies like those of Lam et al. 2014 [11] and Shavell et al. 2012 [12] but in our study we did not find that.

There was no significant difference in the occurrence of adverse effects in pregnancy with single or multiple fibroids in the present study although the power of the number of fibroids on obstetric outcomes in some studies is still divisive. Lam et al. [11] reported a higher rate of preterm delivery among patients with multiple fibroids compared with those with a single fibroid. Likewise, Ciavattini et al. [13] monitored raised preterm delivery, cesarean delivery, and breech presentation rates among individuals with multiple fibroids compared with single fibroids or no fibroids.

However, Qidwai et al. [14] reported no correlation between increased numbers of fibroids and adverse obstetric outcomes and Lai et al. [15] recorded no relationship between preterm delivery and fibroid number.

In our study, vaginal delivery was less than cesarean section. In various studies, rate of cesarean section ranges between 34% and 73%. Klatsky et al. 2008 recorded that women with fibroids were at a 3.7-fold increased risk of cesarean delivery [7]. Vergani et al. 2007 reported that multiple fibroids, large fibroids, and fibroids in the lower uterine segment are predisposing factors for cesarean delivery [16]. Changes in fibroids during pregnancy stay divisive. In the current study, fibroids ≤ 2 cm at first evaluation increased in size whereas fibroids that were ≥2 cm showed no change in size during the second trimester. Benaglia et al. reported significant fibroid growth during early pregnancy and explained human chorionic gonadotropin as an important contributing factor [17]. Lev-Toaff et al. described that fibroids either increased in size or remained unchanged, in response to increased estrogen in the first trimester, and in the second trimester, smaller fibroids (2–6 cm) increased in size or remained unchanged while larger fibroids (>6 cm) decreased in size, maybe due to the starting of estrogen receptor downregulation. Lastly, during the third trimester, fibroids decreased in size or stayed unchanged because of estrogen receptor downregulation [18]. On the other hand, Rosati et al. reported that 69% of pregnant women who had a fibroid practiced no increase in fibroid volume [19]. Laughlin et al. proofed reduction in fibroid size during pregnancy [20]. Consistently, our results somewhat agreed with the findings of Lev-Toaff et al. Nevertheless, it is impractical to expect the growth of fibroids perfectly as fibroids responding to pregnancy in a dissimilar way in different individuals [18]. Moreover, no studies have yet illuminated the effects of several confusing factors on the growth of fibroids in pregnancy.

## 5. Strengths and Limitations of the Study

Our study had* limitations* of being just observational one not having a comparing group, the sample size was small, some popular concepts could have resulted in a high cesarean delivery rate, and all patients included in the study had no submucosal fibroids. The agreement of our results with the previous studies strengthened the current study.

## 6. Conclusion

Pregnant patients who have fibroids were exposed to high incidence of complications throughout antepartum, intrapartum, and postpartum period. So, they have to be carefully screened in the antenatal period through regular follow-up. Most of the fibroids are asymptomatic but may adversely affect the path of pregnancy and labor dependent on their location and size. The broad employment of ultrasonography has simplified diagnosis and management of fibroids in pregnancy.

## Figures and Tables

**Table 1:** Demographic characteristics.

Variable	Number of OR (mean ± SD)	Percentage %
Age	31.80 ± 3.27	
Prepregnancy body mass index (BMI)	24.67 ± 2.46	
*Gravidity *		
Primigravida	11	23.4
Multigravida	36	76.6
*Duration of menstrual cycle/day *	29.68 ± 3.10	
*Duration of menstrual period/day*	6.46 ± 1.12	
Spontaneous conception	59.57%	28
Using assisted reproductive technology	40.43%	19

Values are given as mean ± SD or number (percentage %).

**Table 2:** Maternal outcome during antenatal period.

Outcome	Number (*N*)	Percentage (%)
Threatened miscarriage	16	(34%)
Preterm labor	10	(21%)
Antepartum bleeding—placenta previa	1	(2%)
Abdominal pain needing admission	2	(4%)
Laparotomy due to pain	1	(2%)
Postpartum hemorrhage	2	(4%)
Blood transfusion	1	(2%)

Values are given as number (percentage %).

**Table 3:** Pregnancy outcome.

Outcome	Number (*N*)	Percentage (%)
Spontaneous abortion	1	(2%)
Premature delivery	13	(27.7%)
Delivery at 37–41 weeks	33	(70.2%)
Vaginal delivery	7	(15%)
Cesarean sections	39	(85%)

Values are given as number (percentage %).

**Table 4:** Neonatal outcome.

Outcome	Number (*N*)	Percentage (%)
Congenital anomaly	1	2%
Fetal weight	2978.15 ± 374	
Apgar score		
(i) Apgar score ≤ 7 at 1 minute	2	4%
(ii) Apgar score ≤ 7 at 5 minutes	1	2%
(iii) Apgar score at ≤ 7 at 10 minutes	0	0
Neonatal admission (NICU)	0	0

Values are given as mean ± SD or number (percentage %).

**Table 5:** Obstetric outcomes between patients with single or multiple fibroids.

Outcomes	Patients with single fibroid (*N*)	Patients with multiple fibroids (*N*)	*P* value
Threatened miscarriage (16)	9	7	0.077
Preterm labor (10)	4	6	0.88
Antepartum bleeding—placenta previa (1)	1	0	0.96
Abdominal pain needing admission (1)	1	0	0.96
Laparotomy due to pain (1)	0	1	0.96
Postpartum hemorrhage (2)	1	1	1.0
Blood transfusion (1)	1	0	0.96
Spontaneous abortion (1)	0	1	0.96
Premature delivery (13)	8	5	0.187
Delivery at 37–41 weeks (33)	19	14	0.586
Vaginal delivery (7)	3	4	0.97
Cesarean sections (39)	22	17	0.123

Values are given as number; *P* < 0.05 was considered statistically significant.

**Table 6:** Obstetric outcomes between patients with different types of fibroid.

Outcomes	Patients with intramural fibroid (*N*)	Patients with subserosal fibroid (*N*)	*P* value
Threatened miscarriage (16)	6	10	0.97
Preterm labor (10)	3	7	0.78
Antepartum bleeding—placenta previa (1)	0	1	0.97
Abdominal pain needing admission (1)	1	0	0.96
Laparotomy due to pain (1)	1	0	0.98
Postpartum hemorrhage (2)	2	0	0.841
Blood transfusion (1)	1	0	0.96
Spontaneous abortion (1)	0	1	0.96
Premature delivery (13)	6	7	0.98
Delivery at 37–41 weeks (33)	17	16	0.76
Vaginal delivery (7)	5	2	0.68
Cesarean sections (39)	27	12	0.83

Values are given as number; *P* < 0.05 was considered statistically significant.

**Table 7:** Alteration in size of fibroid during pregnancy.

Time of assessment	Fibroids ≤ 3 cm in diameter at participation	Fibroids ≥ 3 cm in diameter at participation
Assessment at 14–16 weeks of pregnancy	1.96 ± 0.82	3.98 ± 1.16
Assessment at 22–26 weeks of pregnancy	2.48 ± 1.27	4.93 ± 1.05
Assessment at 26–34 weeks of pregnancy	3.54 ± 0.96	5.14 ± 1.23
*P* value		
(i) Comparing 1, 2	0.04	0.74
(ii) Comparing 2, 3	0.81	0.23
(iii) Comparing 1, 2	0.02	0.19

Values are given as mean ± SD; *P* < 0.05 was considered statistically significant.
